# Can a present-day thermal niche be preserved in a warming climate by a shift in phenology? A case study with sea turtles

**DOI:** 10.1098/rsos.221002

**Published:** 2023-02-08

**Authors:** Jacques-Olivier Laloë, Graeme C. Hays

**Affiliations:** Deakin University, Geelong, Victoria 3216, Australia

**Keywords:** climate change adaptation, marine turtles, climatology, conservation, endangered species

## Abstract

How species respond to climate change may impact their extinction probability. Here we link climatology and ecology to tackle a globally important conservation question. For sea turtles, there are concerns that climate warming will cause both the feminization of populations as well as reduced hatchling survival. For 58 nesting sites across the world spanning all seven sea turtle species, we investigated whether warming might be avoided by shifts in nesting phenology to a cooler part of the year. We show that even with the most extreme phenological shift that has been reported to date—an 18-day advance in nesting per °C increase in sea surface temperature (SST)—temperatures will continue to increase at nesting sites with climate warming. We estimate that SST at nesting sites will rise by an average of 0.6°C (standard deviation = 0.9°C, *n* = 58) when we model a 1.5°C rise in SST combined with a best-case-scenario shift in nesting. Since sea turtles exhibit temperature-dependent sex determination, these temperature rises could lead to increasingly female-biased sex ratios as well as reduced hatchling production at sites across the world. These findings underscore concerns for the long-term survival of this iconic group.

## 1. Introduction

The pace at which animals respond and adapt to climate change may be central to their survival [1,2]. There are different means by which species may adapt to a changing environment. For example, rapid evolutionary change offers potential for adaptation for species with short-generation times, such as some plankton [3]. However, when generation times are longer (e.g. several decades), rapid genetic adaptations in the face of climate change are unlikely [4]. Range changes offer another way to adapt to a changing climate and have been seen widely across diverse taxa such as butterflies, plankton, birds and amphibians [5]. For species that are vulnerable to climate change but are unable to undergo rapid genetic adaptation or to change their range, other adaptive measures are needed to avoid local extinctions.

Of the seven extant sea turtle species, six appear on the IUCN Red List of Threatened Species (www.iucnredlist.org): greens (*Chelonia mydas*, globally endangered), hawksbills (*Eretmochelys imbricata*, globally critically endangered), Kemp's ridleys (*Lepidochelys kempii*, globally critically endangered), leatherbacks (*Dermochelys coriacea*, globally vulnerable), loggerheads (*Caretta caretta*, globally vulnerable) and olive ridleys (*Lepidochelys olivacea*, globally vulnerable). Flatbacks (*Natator depressus*) are classified as ‘data deficient’. Climate change is impacting sea turtles in a number of ways throughout their life cycle [6]. Potential impacts range from the loss of nesting beaches due to sea-level rise and increased erosion [7] to changes in oceanic distribution due to the alteration of wind patterns and ocean currents [8]. Increased exposure to extreme thermal events (e.g. marine or air heatwaves) may also impact sea turtle foraging grounds and threaten their reproductive output [9,10]. Sea turtles have also long been considered to be at high risk from climate warming since they have temperature-dependent sex determination [11–13]. Adult females nest on beaches across the world in the tropical, sub-tropical and temperate zones [14]. They lay their eggs in nest chambers dug several tens of centimetres deep and then cover their eggs with sand. No parental care is given thereafter. Incubation typically lasts between 40 and 80 days, depending on the species and incubation temperature (e.g. [15]). Females are produced at warmer incubation temperatures [16], and so there is concern that climate warming might cause the production of highly female-skewed hatchling cohorts, which could ultimately lead to population extinction [6,17]. In addition, lower hatch success at high incubation temperatures threatens population survival [18–20]. Currently across species, most sea turtle nesting beaches around the globe produce hatchling sex ratios that are already heavily female-biased [21]. Recently, the largest green sea turtle rookery in the world was shown to be extremely female-biased [22,23].

Sea turtles have long generation times (several decades), which precludes rapid evolution of the pivotal temperature for sex determination as a means to adapt to climate change. In addition, females exhibit tight fidelity to their nesting area [24], which suggests turtles cannot readily change their range to accommodate warming temperatures [25]. Because of this tight natal fidelity and long generation time, phenological shifts in nesting (i.e. changes in the timing of nesting events) have been widely proposed as the most likely means by which sea turtles could adapt to warming temperatures [26–28]. Marked phenological changes have been observed widely across taxa (e.g. insects, amphibians and birds; [29,30]) and with sea turtles there is empirical evidence that warming temperatures can result in earlier nesting [26,27,31]. However, it is not known if the rate of phenological shifting is sufficient to mitigate future climate warming across species and populations. Here, we investigate how the thermal niche used by sea turtles is likely to change with a combination of climate warming and shifts in nesting phenology. In short, we define the current thermal niche sea turtles use at 58 rookeries across the world and project how these thermal niches would change under a scenario in which sea surface temperatures (SST) rise and turtles nest earlier in the year. We define the thermal niche turtles use with SST measurements recorded adjacent to their nesting beaches.

Previous studies have modelled how SST are likely to vary at different breeding sites within a specified timeframe (e.g. 50, 100 or 200 years) and under various climate warming scenarios (e.g. [28,32]). Here, we present a different conceptual approach and propose the question: can see turtles adapt to a 1.5°C increase in SST by shifting their nesting season to a cooler time of the year? In this manner, we consider whether phenological shifts in nesting could preserve the current thermal niche for sea turtles around the world, regardless of which climate warming scenario is followed. We projected a 1.5°C increase in SST, as this warming is very likely to happen before the end of the century (e.g. under ‘Middle of the Road’ scenario SSP2–4.5 it is projected that SST will increase by 1–4°C globally by 2081–2100 relative to 1850–1900; [33,34]).

## 2. Material and methods

### 2.1. Conceptual framework

**Figure 1.**
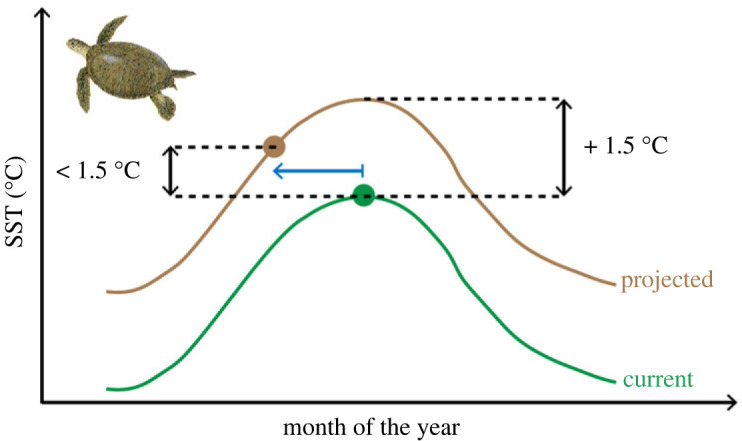
Conceptual framework for how a rise in SST may be mitigated by a phenological shift of the nesting season. The green and brown lines represent the current and projected SST. The filled circles represent the peak of the sea turtle nesting season. In this case, a projected 1.5°C rise in SST translates to less than 1.5°C rise in SST during peak nesting season due to a phenological shift to earlier nesting (blue arrow). The turtle image was kindly provided by NOAA Fisheries (www.fisheries.noaa.gov).

We conceptualized how rising SST and a shift in nesting phenology might interact to impact future conditions (figure 1). Here we describe the conceptual framework in four steps. Details for different steps are given thereafter in §2.2.

1. We consider a seasonal cycle of SST adjacent to a sea turtle nesting area.
2. We increase the SST at each site by 1.5°C.
3. We shift the nesting phenology forward by 27 days (i.e. the most extreme phenological shift reported in the literature for a 1.5°C rise in SST).
4. We observe the difference between the projected and current SST during peak nesting season. If this value is less than 1.5°C we consider that part (or all) of the warming was mitigated due to the phenological shift (figure 1).

**Figure 2.**
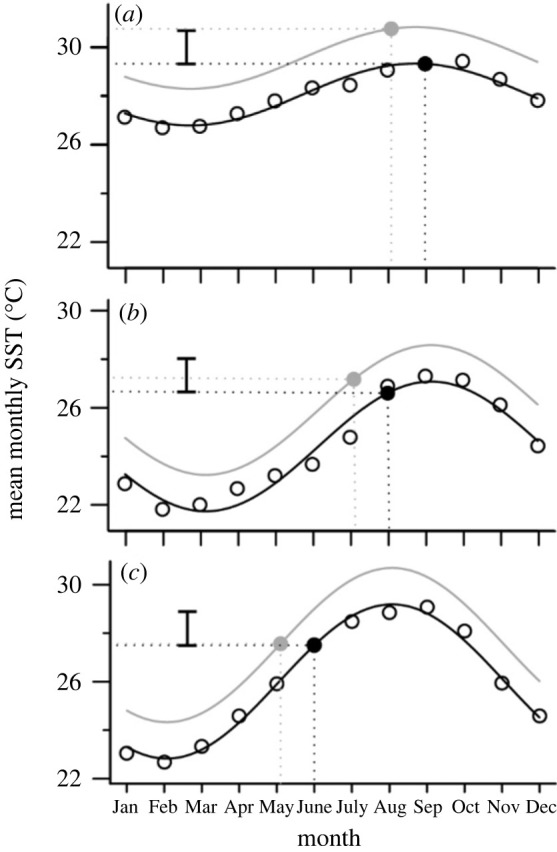
Phenological shifts of the nesting season have variable impacts at different sites. We modelled how a 1.5°C rise in SST combined with a 27-day advance of the nesting season would impact SST at 58 sea turtle nesting sites. Here we highlight three case examples: (*a*) On Saint Eustatius in the Caribbean, a shift of the nesting season does not mitigate any warming SST. (*b*) On Sal in the Northeast Atlantic, a shift of the nesting season mitigates approximately 60% of a 1.5°C rise in SST. (*c*) In Florida in the Northwest Atlantic (*c*), almost 100% of warming is mitigated by a best-case-scenario phenological shift. Open circles represent mean monthly SST and the black line represents the sine fit. The grey line represents projected conditions after a 1.5°C rise in SST. The filled circles represent a month during the peak of the nesting season in their respective scenarios. For easy comparison between subpanels, the vertical bars represent 1.5°C. The geographical location of these three study sites is highlighted in figure 3.

**Figure 3.**
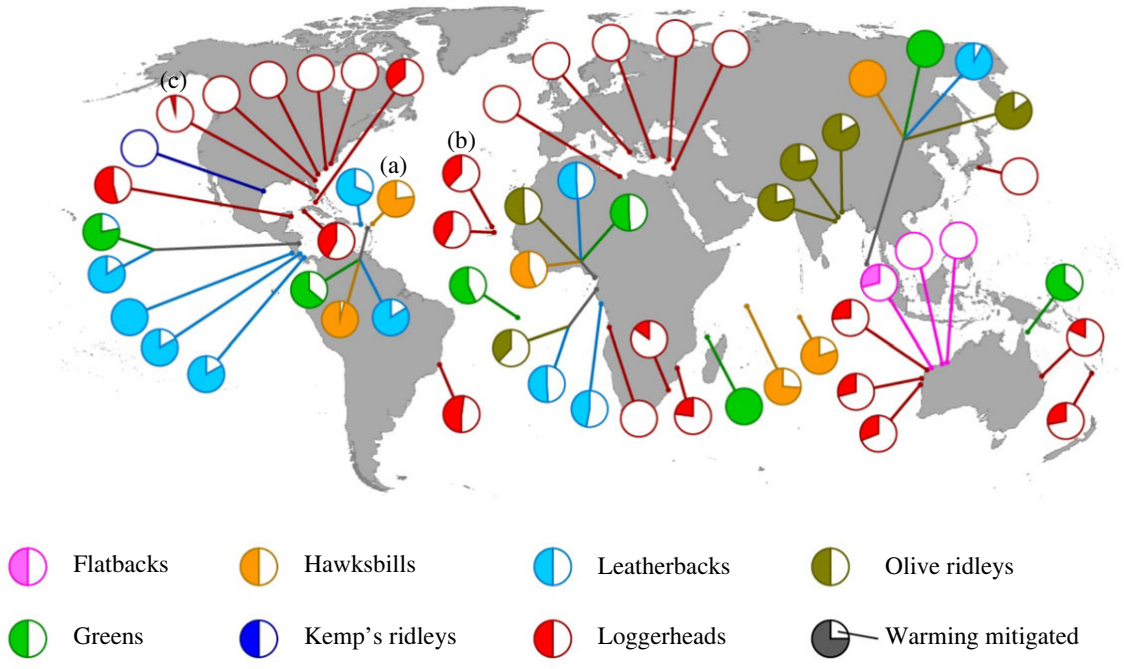
Global patterns for the interaction of rising SST and shifting nesting phenology. We show how a 27-day shift of the nesting in response to a 1.5°C rise in SST would affect SST at all 58 sites used in our study. The filled slice of each pie chart represents the proportion of the 1.5°C rise in SST that occurred, such that a completely full pie indicates that no warming is mitigated. Colours represent the different turtle species. The sites highlighted in figure 2 are indicated here: (*a*) Saint Eustatius (the Netherlands) in the Caribbean, (*b*) Sal (Cape Verde) in the Northeast Atlantic and (*c*) Florida (United States of America) in the Northwest Atlantic.

**Figure 4.**
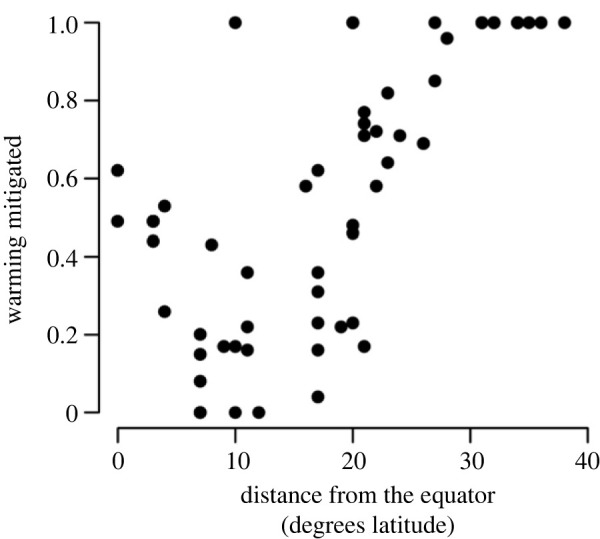
Phenological shifts in nesting at sites farthest from the equator have more impact on mitigating warming SST. The proportion of warming that was mitigated is plotted across sites. A 27-day shift in nesting in response to a 1.5°C rise in SST was most effective at maintaining current SST at sites greater than 30° latitude

This combination of increased SST and phenological shift in nesting can potentially lead to an increase, decrease or no change in SST, depending on the nature of the seasonal change in SST and nesting seasonality. Using this conceptual framework, we parameterized the interaction of climate warming and a phenological shift in nesting for 58 sites around the world (figures 2–4).

### 2.2. Empirical datasets

#### 2.2.1. Sea surface temperature

We obtained SST from the International Comprehensible Ocean-Atmosphere Data Set (ICOADS) through the National Center for Atmospheric Research (http://rda.ucar.edu/datasets/ds540.1/). The ICOADS is an extensive surface marine dataset compiled from different monitoring systems, including coastal meteorological stations, moored buoys, research vessels and surface drifters. We used the Enhanced ICOADS Monthly Summary Statistics Release 3.0.0 to obtain SST for the 2° by 2° quadrats that encompass relevant sea turtle nesting sites (i.e. the selected quadrats contained the nesting beaches as well as the area adjacent to the beaches). If a turtle rookery consisted of more than one nesting beach, we used the nesting beach with the largest turtle aggregation as the reference point around which to place the quadrat (electronic supplementary material, table S1). We used mean monthly SST recorded between January 2009 and December 2019 to describe recent annual fluctuations in SST at each nesting site. A sine function was fitted to these mean monthly SST to model year-round SST (figure 2).

To model future climate warming, we increased modelled SST by 1.5°C (figure 1) since the Intergovernmental Panel on Climate Change (IPCC) warns that a 1.5°C increase in SST is very likely to happen before the end of the century [33,34]. We compared current SST experienced during the peak of the nesting season (see point 2.2.2 Nesting seasonality, below) to projected SST after a 1.5°C increase in SST and corresponding phenological shift to earlier nesting were modelled to establish if any warming was mitigated due to the phenological shift (figure 1).

#### 2.2.2. Nesting seasonality

We extracted nesting seasonality data and coordinates of as many sea turtle rookeries as possible from literature sources (electronic supplementary material, table S2). Sources included research articles published in peer-reviewed journals, reports from the International Union for Conservation of Nature and Natural Resources (www.iucn.org) and reports published by The State of the World's Sea Turtles (www.seaturtlestatus.org). When different seasonality information was given for different sites, we used the most recent source. We searched for data for all seven extant sea turtle species—flatbacks, greens, hawksbills, Kemp's ridleys, leatherbacks, loggerheads and olive ridleys—including small nesting aggregations (e.g. leatherbacks nesting in Saint Eustatius, the Caribbean) as well as some of the world's largest rookeries (e.g. greens nesting in Raine Island, South Pacific). If the peak of the nesting season was not given in the publication, we assumed that the peak occurred in the middle of the nesting season, since sea turtle nesting seasons generally follow a relatively symmetric bell-shaped pattern [35].

We defined the nesting seasonality and SST for 62 nesting sites around the world, including all seven species of sea turtles (electronic supplementary material, table S2). A sine function fitted to mean monthly SST recorded between January 2009 and December 2019 described annual fluctuations in SST well at 58 out of the 62 nesting sites (i.e. the model's *p*-value was less than 0.05; electronic supplementary material, figure S1). We excluded from our analysis the four sites with a poor model fit (i.e. four out of 62 sites). We checked normality of the residuals through visual inspection of the residual plots. Inter-annual variability was homogeneous between months and so did not affect the model's output.

#### 2.2.3. Phenological changes

We searched the literature for publications that report phenological shifts in nesting sea turtles (electronic supplementary material, table S3). We entered the search terms ‘phenological shift’ and ‘sea turtle’ as TOPIC in Web of Science (apps.webofknowledge.com). We did a backward and a forward citation search on relevant articles to find further articles. To provide the most optimistic scenario for how a phenological shift in nesting might help mitigate climate warming, we used the most extreme published relationship for the link between SST and nesting dates—an 18-day advance in nesting per 1°C increase in SST reported for loggerheads (electronic supplementary material, table S3), which translates into a 27-day advance of the nesting season for a 1.5°C rise in SST in our study.

## 3. Results

### 3.1. Sea surface temperature and nesting seasonality

In the majority of cases, sea turtles nest during the warmer months of the year (electronic supplementary material, figure S1). The northernmost nesting sites in our study were Zakynthos in Greece and Fethiye in Turkey, and the southernmost nesting site were KwaZulu-Natal in South Africa and Dirk Hartog Island in Australia (figure 3). 25 of the sites used in our study are loggerhead nesting sites, ten are leatherback sites, seven are green sites, six are hawksbills, six are olive ridley sites, three are flatbacks, and one is a Kemp's ridley nesting site (i.e. the only nesting site colonized without human intervention; figure 3).

### 3.2. Phenological changes

We found 18 relationships between SST and phenological shift from nine publications (electronic supplementary material, table S3). Relationships were given for loggerhead, leatherback and green turtles nesting at seven different sites. The longest study period was 25 years on loggerheads nesting on Zakynthos, Greece, between 1984 and 2009 [36]. The most extreme phenological change observed was an 18-day advance in nesting per 1°C increase in SST and was reported at two sites: Zakynthos, Greece [37] and Bald Head Island, United States of America [38]. We used this value to present the most optimistic scenario for how a phenological shift in nesting might help mitigate climate warming. In our study, since we are projecting a 1.5°C rise in SST, the resulting best-case-scenario phenological shift would be of 27 days.

### 3.3. Warming mitigated

The interaction of a 1.5°C warming and a 27-day phenological shift in nesting was variable across sites (figures 2–4). For example, a 27-day advance of the nesting season was unable to mitigate 1.5°C SST warming on Saint Eustatius (in the Caribbean; figure 2*a*). By contrast, on Sal (in the Northeast Atlantic), the same phenological shift in nesting mitigated 62% of a 1.5°C rise in SST (figure 2*b*). In Florida (Northwest Atlantic), nearly all warming was avoided by the same phenological shift (figure 2*c*). Note that we are referring here to the Peninsular Florida subpopulation (a loggerhead subpopulation defined by Loggerhead Turtle Expert Working Group), which includes nesting beaches that are geographically close and have the same nesting season ([39]; electronic supplementary material, table S1).

Rising SST were entirely mitigated by phenological shifts at 14 sites, while at four sites no warming was mitigated (figure 3). For all 58 sites, phenological shifts mitigated on average 55% of warming SST (standard deviation = 34%, min = 0, max = 100, *n* = 58; electronic supplementary material, table S4).

In addition, we found a broad latitudinal effect (logistic fit, *p* < 0.05, *r*^2^ = 0.49, *n* = 58), with phenological shifts at higher latitudes having more impact on mitigating warming temperatures (figure 4). This effect likely occurs because of the more marked seasonality in SST at higher latitudes (electronic supplementary material figure S1).

## 4. Discussion

Climate change is having many ecological impacts, including changes in species ranges and the phenology of seasonal events such as migration and breeding [30,40,41]. While many of these changes are well described, much less clear is whether taxa can preserve their current thermal niche through the interaction of rising temperatures with changes in range or phenology [42,43]. Yet the outcome of this interaction may have strong implications for species survival, particularly when species are already threatened by various anthropogenic impacts such as habitat loss and harvesting. Our findings suggest that, all else being equal, even the maximum reported rate of phenological shift in nesting will often not be enough for sea turtles to fully mitigate rising temperatures occurring as part of climate change. Our results extend and reiterate the conclusions from previous studies at a few key sites [28,44]. Here we offer a global view of this research topic that includes all seven species of sea turtles. Additionally, a result emerging from our analysis is that the impact of a phenological shift in nesting will vary around the world, and that at higher latitudes more of the future rises in SST will likely be mitigated by phenological changes. Taken together, a key message is that we cannot assume that turtles nesting around the world will always be able to naturally mitigate climate warming impacts by a phenological shift in nesting.

Most concerning is that we explored a best-case-scenario, so it is likely that sea turtles have less adaptive potential than presented here. We likely provide an overly optimistic view of the impact of a shift in nesting, since we used the maximum reported rate for this phenological shift. Running the same analysis using the average phenological shift reported in the literature (i.e. a 9-day advance in nesting per °C increase in SST; electronic supplementary material, table S3) reveals that SST at nesting sites will rise by an average 1.0°C (standard deviation = 0.4°C, *n* = 58). Using a 4-day advance in nesting per °C increase in SST (as reported in 2009 by [45]) results in SST rising by an alarming 1.4°C (standard deviation = 0.1°C, *n* = 58).

It may be that different species of sea turtles are not able to respond to warming temperatures in the same way. Relationships between phenological shifts in relation to sea surface temperatures were available from seven different sea turtle nesting sites (electronic supplementary material, table S3) but only for three species (i.e. loggerheads, leatherbacks and greens). Furthermore, at some sites there was no evidence for earlier initiation of the nesting in response to warmer temperatures [46]. More data are needed to better inform how different species are likely to fare in the face of climate warming. In addition, some sea turtle populations already nest at the coolest time of the year (e.g. flatback turtles nesting at Cape Domett in Australia; [47]) so a shift in the phenology of nesting will not mitigate increases in SST at these nesting locations. Indeed, a shift in phenology of nesting—if it occurred—would increase temperatures even more.

Rising SST are a threat to sea turtles because of the close relationship between SST and sand temperature, i.e. the environment in which sea turtle eggs incubate. Previous work has shown that SST and air temperature (AT) measured over large scales near nesting sites are tightly correlated with sand temperature at nest depths [48–51]. More recently it was shown that the gradients of these relationships are consistent across sites, and that for every 1°C increase in AT, sand temperature at nest depth increases by an average 0.86°C (standard deviation = 0.26°C, *n* = 36; [52]). Similarly, for every 1°C increase in SST, sand temperature increases by an average 0.72–0.83°C [48,52]. In other words, because of the tight positive relationship between SST and sand temperature it is very likely that if SST rises at one site, so will sand temperature at nest depth. In general terms, a 1.5°C rise in SST—as modelled in our analyses—would translate into approximately 1.1°C rise in sand temperatures at nest depth, although the absolute values are likely to vary slightly between sites. Due to the steep relationship between incubation temperature and hatchling sex ratio [53], a 1.1°C difference in nest temperature can be the difference between an all-male and an all-females nest, so 1.1°C is not a negligible value.

At sites around the world, it will be important to maintain empirical measurements to detect climate warming impacts on incubation conditions and so we emphasize the importance of long-term monitoring as is done in many ecosystems [54]. There are various ways in which the signs of excessive feminization might be detected. First, direct measurements of hatchling sex ratios or sand temperature at nest depths may show long-term feminization or warming respectively. Since direct measurements of sex ratios are fatal, an alternative is to estimate hatchling sex ratios using temperature-based models [55]. At sites where long-term measurements of sex ratios are not available, indications of warming might still be evident since very female-biased hatching sex ratios are accompanied by high in-nest embryo mortality [19,56] and lower hatchling quality [57–59]. Consequently, simple measures such as hatchling success (the proportion of eggs developing into hatchlings) may provide an alert to feminization that could then be substantiated by targeted measurements of hatchling sex ratios.

Because of the negative effects of warming temperatures on hatchling production and sex ratios, methods to reduce sand temperatures at nesting beaches are being explored. Strategies to artificially cool nests, such as through shading or watering, have already been trialled on different nesting beaches and offer promising results [20,60–62]. For example, shading was shown to decrease sand temperatures by an average 0.60°C on a nesting beach in Saint Eustatius (figure 2; [60]). Similarly, irrigating artificial green sea turtle nests with seawater or freshwater resulted in an immediate 1.3°C drop in nest temperatures on Heron Island (Australia; [63]). Such strategies could be implemented at sites where excessive feminization or hatchling mortality is occurring but require careful consideration. For example, there are concerns that lowering nest temperatures would alter sex ratios in a detrimental way, since having fewer females would reduce future population reproduction output [20,64]. Therefore these mitigation strategies should not only consider short-term benefits (e.g. increased hatchling production) but also promote positive long-term outcomes (e.g. population recovery or stability). Finally, it is important to note that such mitigation strategies would only offer a temporary ‘Band-Aid’ solution, as the underlying issues of climate change are not being addressed. Solutions to address climate change, like reducing greenhouse gas emissions, switching to renewable energies, and changing land-use patterns are required to reduce future climate change impacts.

There are clearly caveats to any predictions for how ecosystems will change in the face of climate warming, and this applies universally and not just to SST as seen in our study [65–67]. Our methods could be refined in various ways, such as by considering likely temperatures across entire nesting seasons rather than at just one point. Access to local SST datasets with better resolution and accuracy could also improve the model's output. Since variability in thermal niches exists within nesting grounds, our broad approach could also be refined for sites with inter-annual and intra-beach temperature data available. There also remain a number of uncertainties for how climate change will impact temperatures at sea turtle nesting sites. For example, there are uncertainties for how patterns of rainfall will change in the future [68,69]. In some nesting sites it has been shown that heavy rainfall may be sufficient to cool the sand to the extent that more male hatchlings are produced [61,70,71], so one scenario is that increased heavy rainfall may help mitigate climate warming. Second, it is possible that sea turtles might colonize new cooler nesting sites in the future. Generally, turtles have tight fidelity to their natal nesting areas, with flipper tagging showing turtles returning to broadly the same area to nest [24,72]. However, occasional breakdowns in fidelity are recorded [73–75] with, exceptionally, turtles nesting at sites 100s of km apart (e.g. [76]). Furthermore, when nesting beaches become less suitable, e.g. through increased light pollution due to development or beach erosion, turtles may shift nesting to nearby beaches [77]. It is possible that these breakdowns in fidelity could have helped with adapting to past climatic cycles, as a species' range could have expanded and contracted spatially as groups or individuals departed from philopatry. However, it remains unknown if the speed at which new sites are colonized will be sufficient to prevent feminization in nesting populations in the face of warming.

Finally, climate warming may not apply equally across seasons [78,79] and so our calculations could be refined as more information emerges on likely site-specific changes in SST seasonality. Elegant mechanistic approaches have been used to assess the drivers of incubation temperatures [47,80] and although these models are still susceptible to uncertainties on how environmental conditions such as rainfall may change [6], they provide another approach to assessing likely future changes in temperatures at sea turtle nesting sites. While both correlative and mechanistic models produce very similar results [50,81], the mechanistic approach has the advantage that it can be forced with novel combinations of environmental variables to explore different climate change scenarios. Unfortunately, it was not possible to use microclimate models in our study since the input parameters (e.g. beach properties and local meteorological data) required to run the models were not available for all study sites. However, microclimate models offer great potential in this area and may help shed further light on expected changes in incubation temperatures in the face of climate warming [47,50].

Our approach to examine how phenological shifts could mitigate climate warming can be applied broadly to other species faced with adapting to climate change. For example, our methods could be applied to other organisms that exhibit temperature-dependent sex determination, such as crocodiles and tortoises [82]. Similarly, it would be possible to assess if the rate of phenological change some migratory species exhibit would allow them to maintain their thermal niche as seasonal cycles change [83]. Since the data needed to apply our methods— i.e. temperature records, nesting seasonality data, and phenological change rates—are often commonly monitored as part of conservation and research, applying our methods could be easily done for a wide range of study organisms.

While uncertainties remain for the likely impacts of climate warming on sea turtles, our results add to the growing evidence that a phenological shift in nesting will often be insufficient, by itself, to maintain current thermal conditions [28,44]. Future work might consider how a range of processes (e.g. geographical range shifts) may buffer climate-warming impacts for sea turtles.

## Data Availability

The data are provided in electronic supplementary material [84].
